# Supplementary material for: Are carbon emissions trading and green financial instruments synergistic? -Comprehensive quantitative research based on content analysis
Source: PLoS One. 2024 Mar 7;19(3):e0298601. doi: 10.1371/journal.pone.0298601 (PMC10919601; doi:10.1371/journal.pone.0298601)
Supplement: S1 Data — (ZIP) [file pone.0298601.s001.zip › Data/地级市Z_F工作报告.zip!/║■▒▒/╦μ╓▌╩╨/└┤╫╘╣1⁄2╓┌║┼í╛┬φ┐╦╩2╛▌═°í┐_2011.docx]

各位代表：

现在，我代表市人民政府向大会报告政府工作，请予审议，并请市政协委员和各位列席人员提出意见。

一、2010年工作回顾

2010年，市政府在省委、省政府和市委的坚强领导下，始终坚持以科学发展观为统领，深入贯彻落实党的十七届四中、五中全会精神，着力稳增长、调结构、惠民生，全面完成市二届人大五次会议确定的各项任务。

——综合实力快速提升。全市经济持续快速发展，较好保持了超过年初预期、高于全省和全国平均水平的“一超两高”良好态势。地区生产总值突破400亿元，较上年增长14%；实现规模工业增加值140亿元，同比增长26%；完成财政总收入21.96亿元、同比增长25.8%，其中地方一般预算收入9.53亿元、增长25.5%。

——工业结构不断优化。以专用汽车及零部件为主导，支柱产业加速扩张，汽车机械产业实现增加值42亿元，同比增长26.8%。中国恒天、湖北三环、厦门重工等一批大企业相继落户；齐星公司、全力铸造、华龙车灯、茂鑫胶带等一批本土企业快速扩张，齐星公司实现车身产量6.45万台、同比增长1倍多，销售收入17亿元、同比增长60%，投资13亿元、历时2年多建设的多晶硅项目顺利投产；中国机械工业联合会与省政府签署共建中国专用汽车之都合作协议，都极大地提升了我市专汽产业的知名度和竞争力。医药化工、纺织服装、电子信息、风机制造产业加快发展，特别是食品工业增势强劲，实现增加值30亿元、同比增长28%，成为第二大支柱产业。“中小企业成长工程”成效显著，亿元企业和新增规模企业双双突破100家，净增规模企业52家。积极推进科技进步与创新，产学研合作深入开展，建成2个省级工程技术中心，新增高新技术企业21家。企业上市步伐进一步加快，9家公司被列入上市后备企业。

——农业基础更加稳固。农业生产战胜洪灾、旱灾和虫灾的侵袭，连续8年喜获丰收。粮食总产达到155.7万吨，增产5.7万吨。特色产业板块继续扩大，油茶达到10万亩，培植了2个万亩镇、24个千亩村；食用菌出口突破3亿美元，居全国地级市首位。农产品品质不断提高，大洪山牌香菇、集蜂堂牌蜂蜜荣获全省十大名牌农产品称号。农业产业化步伐进一步加快，新增2家省级龙头企业，曾都区的农业加工园区被列入省重点支持园区。农田水利基本建设加快实施，完成53座水库的除险加固。积极推进生态家园建设，长岗镇、吉祥寺村分别被授予“省级生态乡镇”和“省级生态村”的称号。随南、广水北三镇新农村试验区加快建设，村庄整治成效明显。

——文化旅游实现突破。以建设旅游经济强市和“西圈”门户城市为目标，全力打造节会品牌，成功举办2010世界华人炎帝故里寻根节，炎帝故里景区跻身鄂西十大旅游景区之列。投资13亿元，开工文化旅游项目75个，炎帝故里核心景区、玉龙温泉二期等工程顺利完成，大洪山慈恩寺、玉龙漂流等项目抓紧实施；重点景区、旅游名镇名村创建工作得到加强。全市接待游客590万人次，旅游综合收入38亿元，同比分别增长23%和37%。加快改造提升传统服务业，新建了一批网点和物流配送中心，浙江银泰集团与新世纪购物中心的战略重组顺利完成。全年实现社会消费品零售总额198亿元，同比增长20%。深入开展农资、成品油、烟草、食品药品等专业市场整顿，市民消费安全得到进一步保障。

——县域经济竞相发展。不断加大支持力度，县域经济活力进一步增强，有力支撑了全市发展稳定大局。随县招商引资和项目建设呈现强劲态势，“一区四园”加快推进，新县城建设全面启动；广水市三大百亿园区初具规模，华鑫冶炼、西门电机等一批项目相继落户、竣工投产，工业税收除烟厂之外首次突破亿元；曾都区实施百日攻坚，推进二次创业，工业和投资增幅均高于全市和全省平均水平。

——发展后劲得到提升。投资保持强劲增长。全年完成全社会固定资产投资285亿元，同比增长36.5%。重点基础项目建设加快推进。波导大桥如期建成，新建通村公路661公里，西气东输二线随州段即将通气，麻竹高速随州东段建设快速推进，随信铁路、麻竹高速随州西段、十岗至厉山一级公路前期工作进展顺利。园区承载力得到增强。市开发区先后完成城东工业园 “七通一平”、华裕工业园基础设施等重大工程，316国道城区段包装改造如期竣工；新引进企业22家，完成投资23亿元。在全省144个省级开发区综合实力考核中，市开发区居第5位、曾都开发区居第27位，同时受到省政府表彰。金融瓶颈制约有效缓解。加强政企、银企对接，省市金融机构对我市授信340亿元，创历史新高。至12月底，全市金融机构贷款余额为172.5亿元，比年初增加48.44亿元，增长39%，增幅居全省第一。我市被省政府授予A级金融信用市州，综合考核全省第一。

——城乡面貌持续改观。继续完善城市功能，全面构筑城东新区路网，青年东路、新火车站站前大道和编钟大道投入使用；城南新区神农大道延伸、迎宾大道和炎帝大道改造全面完成；老城区氵厥水西大堤整治工程基本完工，改造背街小巷57条，硬化维修道路2.7万平方米。加强城市规划执法，强制性拆除违法建设1万多平方米；着力整治出门出窗占道经营，积极解决城区高峰时段交通拥堵问题。启动第二批“百镇千村”示范工程，打造了洪山、洛阳、武胜关等一批特色集镇，村镇面貌不断改观。

——改革开放纵深推进。实施开放先导战略，多途径扩大招商引资，全年新引进投资过千万元的项目128个，实际利用内联资金61亿元，同比增长34%；实际利用外资4800万美元，同比增长9%。大力扶持出口龙头企业，出口过千万美元的企业达到14家，外贸备案企业新增30多家，全年出口达到7.2亿美元，总量跃居全省中等城市第二位。圆满完成市政府机构改革，公共事业的市场化改革稳步推进。启动国有林场改革，集体林权制度主体改革基本完成。

——人民生活不断改善。实现农民人均纯收入6279元，同比增加822元、增长15.1%；城市居民人均可支配收入15280元，同比增加1819元、增长13.5%。统筹做好重点对象和困难群体就业工作，培训农村劳动力1.64万人，转移就业3.3万人；城镇新增就业1.6万人。发放个人创业和劳动密集型中小企业小额担保贷款3600万元，新增市场主体12600多家、日均增加35家。不断扩大社会保障覆盖面，五项保险新增扩面3.2万人，曾都区新农保试点工作扎实推进，原城镇集体企业职工养老保险全面启动，企业退休人员养老金、城镇职工和居民医疗保险报销比例及支付限额都大幅提高，新农合参合率达到97.7%。住房公积金新增归集1.45亿元，同比增长47%；发放个人住房贷款3639万元，同比增长168%。投入财政资金1100多万元，整合社会资金1500多万元，着力改善贫困地区生产生活条件。解决了33.6万人的饮水安全问题。按计划实施整村推进扶贫工作，驻村扶贫活动深入开展，完成1万多农村人口的脱贫目标。救灾救济及时有效，共恢复重建因灾倒损民房6700多户、1.6万间。在全省率先完成丹江口库区移民安置任务，受到移民称赞、省政府表彰。突出解决困难群体的住房问题，新增廉租住房租赁补贴3498户，建成廉租住房786套，提供公共租赁房680套，改造农村危房500户。年初政府承诺的10件实事全面完成，广大群众关心的一些急难问题，得到一定程度缓解。

——社会事业全面进步。坚持优先发展教育，加快寄宿制学校建设，教育质量继续提高，曾都区、随县跻身省义务教育均衡发展实验区县，随州职院、市技师学院双双被授予国家职业技能培训示范基地。统筹抓好医疗卫生工作，曾都区基本药物制度试点改革进展顺利，药品全部实行零差价销售；投资9000多万元，高标准推进城乡基层医疗卫生机构建设，广水市疾病预防控制经验在全国推广；重大传染病和疫情防控切实有效，成功处置了各类突发公共卫生事件。大力发展文化体育事业，第三次文物普查有序推进，新闻出版事业得到加强；新建和改造了14个乡镇综合文化站、362家农家书屋和60处农民健身工程，完成10个社区文化活动室配套建设；广播电视覆盖率达到98.3%，农村免费放映电影1万多场次；积极参加省第十三届运动会并取得较好成绩。文明城市、文明村镇、文明单位等精神文明创建活动蓬勃开展。人口和计生工作继续保持低生育水平，出生人口性别比趋向正常；国土资源管理得到加强，第二次农村土地调查通过省级验收；第六次人口普查进展顺利。省级环保模范城市的创建规划通过省级专家评审，节能减排完成省定目标。高度重视社会稳定，深入开展“平安随州”创建活动，建立健全社会治安综合治理体系，严厉打击各种违法犯罪行为，“五五”普法通过国家检查验收。建成市信访接待服务中心，开通书记、市长专线电话，畅通群众诉求渠道。安全生产继续保持总体稳定态势。供水、供电、通讯、无线电管理和气象工作得到加强，工商、质量技术监督工作取得新成绩。人民防空工作受到国家表彰。国防动员和民兵预备役建设迈出新步伐，创建新一轮省级“双拥模范城”通过考评验收。对台交流合作出现新局面。地方志、民族宗教、外事侨务、妇女儿童、老龄工作和残疾人事业继续较快发展。

一年来，我们高度重视加强政府自身建设，自觉接受人大、政协和社会各界的监督，办理人大议案和建议56件、政协提案137件，办理质量进一步提高。深入开展行风评议和“三评两查”活动，认真落实党风廉政建设责任制，扎实推进惩治和预防腐败体系建设，初步建成行政服务网络平台和电子监察系统，招投标综合监管“五统一”体系建设得以整体推进。

2010年各项目标任务的全面完成，标志着我市“十一五”规划圆满收官。回顾和总结五年的发展历程，事实表明：

过去的五年，是综合实力大幅提升的五年。地区生产总值实现了翻番；地方财政一般预算收入年均增长达到17.5%，2010年是“十五”末的2倍多；主要经济指标增幅连年高于全省和全国平均水平。特色工业、板块农业加快壮大，工业主导地位得到加强，农业基础地位不断巩固，成功摘取中国专用汽车之都、中国香菇之乡等金字招牌。

过去的五年，是发展后劲显著增强的五年。全社会固定资产投资年均增长40%，五年累计达到814亿元，是“十五”期间投资总额的4倍多。规模工业企业总数达到598家，比“十五”期末增加182家。交通建设突飞猛进，累计完成高速公路通车里程183公里、通村公路5100公里。

过去的五年，是城乡面貌深刻变化的五年。城南、城东新区拉开框架，白云湖水库、滨湖风光带等一批重大项目相继建成，新区建设初具规模；老城区基础设施持续改善，绿化、亮化、净化水平明显提升。新老城区互通格局初步形成。成功创建中国优秀旅游城市和省级文明城市、卫生城市、园林城市，荣获省城市规划建设管理“楚天杯”；成功举办两届炎帝故里寻根节和首届编钟文化节。新农村建设扎实推进，农村生产环境、生态环境和人居环境都得到较大改善。

过去的五年，是改革开放阔步前进的五年。大部制改革、四项制度和供销社建设等多项工作创造了随州特色，走在全省乃至全国的前列。招商引资成效显著，累计到位内联资金200多亿元，实际利用外资1.83亿美元；坚持不懈地开拓国际市场，外贸出口逆势上扬，年均增长38%，累计出口18.8亿美元，随州已成为全省重要的外贸出口大市。

过去的五年，是人民群众得到实惠较多的五年。牢固树立以民为本的执政理念，不断完善义务教育经费保障机制，随州职院建设初具规模并通过教育部评估验收。城乡医疗服务能力和水平显著提高，就业和社会保障体系基本健全，五年累计实施61件重大民生工程和实事项目，城乡居民更多地享受到了改革发展的成果，感受到了党和政府的温暖！

在充分肯定成绩的同时，我们也清醒地看到，我市经济社会发展和政府工作中依然存在一些薄弱环节：一是经济总量仍然偏小，综合竞争实力有待进一步增强；二是“一业独大”的工业经济格局仍然没有根本改变，产业结构有待进一步优化；三是社会事业欠账仍然较多，推进和谐随州建设的力度有待进一步加大；四是少数部门办事效率不高的现象仍然存在，发展环境有待进一步改善。对此，我们将采取得力措施，认真加以解决。

各位代表！2010年及过去五年所取得的成绩，是省委、省政府正确领导的结果，是市委总揽全局、科学决策的结果，是市人大、市政协加强监督、大力支持的结果，是全市人民和衷共济、顽强拼搏的结果！在此，我代表市人民政府，向各位人大代表、政协委员，向全市各条战线上辛勤工作的广大干部群众，向驻随部队、武警官兵和政法干警，向各民主党派、工商联、人民团体和驻随单位，向所有关心支持随州发展的老领导、同志们、朋友们表示衷心的感谢，并致以崇高的敬意！

二、“十二五”时期经济社会发展的建议

“十二五”时期，是随州实现跨越式发展的关键时期。这一时期，不仅自身发展的压力较大，而且外部环境更趋复杂，区域竞争更加激烈，因此，各项工作必须立足于以跨越式发展的方式来加以推进。实现随州跨越式发展，既是发挥区位优势、实现后发赶超的客观要求，也是破解发展难题、转变发展方式的必然选择；既是社会各界的热切期盼，也是政府自我加压的一种精神状态和工作追求。

按照实现跨越式发展的要求，“十二五”时期政府工作的指导思想是：以邓小平理论和“三个代表”重要思想为指导，全面贯彻落实十七届五中全会精神，以科学发展观为统领，以工业兴市为主战略，以结构调整为主线，以重大项目为支撑，以改革开放为动力，着力培育特色产业，着力推进城乡统筹，着力转变发展方式，着力保障和改善民生，力争综合实力在全省晋档进位，努力把随州建设成为“鄂西圈”的重要门户、“两圈”融合的重要结合部、全球华人谒祖圣地、全国有影响的优秀旅游目的地和集散地；建成名副其实的中国专用汽车之都、全省最大的农产品出口基地、全省重要的光伏产业基地；建成襄十随城市群重要的枢纽城市、物流中心和山水生态宜居城市。

经济社会发展的主要预期目标是：经济总量迈上新台阶，力争实现“两个高于”和“六个翻番”，即主要经济指标高于“十一五”增长水平、高于全省平均增长水平，地区生产总值、全社会固定资产投资、规模工业增加值、财政一般预算收入、社会消费品零售总额和外贸出口实现翻番。其中，地区生产总值年均增长13%以上，农民人均纯收入年均增长10%以上，城镇居民人均可支配收入年均增长11%以上；各项社会事业全面协调发展。围绕新目标，采取新举措，推进新跨越。

（一）全力扩大经济总量，在转变发展方式上实现新跨越。坚持不懈地推动经济结构战略性调整，致力于构筑多点支撑的现代产业格局，到“十二五”末，力争一二三产业之比调整为15：47：38。

加快提升工业主导地位。突出特色发展，整合资源、优化结构、培植龙头、拉长链条、打造品牌，着力提升核心竞争力，努力构建以专用汽车及零部件、风机制造、农产品加工为主导，特色鲜明、优势明显、贡献突出的地方工业体系。突出创新发展，坚持用高新技术和先进适用技术改造提升化工、建材、冶金、医药、包装等传统产业，大力发展新能源、新材料、节能环保、电子信息、生物技术等战略性新兴产业，着力培植新的经济增长点。突出开放发展，坚持用开放的思维抓工业，着力引进大集团、大项目，加快企业重组、联合和上市步伐，促进优势产业快速扩张。突出集聚发展，坚持以建设先进制造业基地为目标，按照“资源节约型、环境友好型”的要求，加快开发区和各类特色工业园区建设，引导企业向园区集中、生产要素向优势企业聚集，加快产业集群化发展，力争“十二五”末市经济开发区产值过300亿元、冲刺500亿元，曾都经济开发区产值过200亿元、广水和随县经济开发区产值过100亿元，增强县域经济综合竞争力。

加快提升现代农业。纵深推进农业产业化经营，大力发展农产品加工业，加快实施“四个一批”和“一袋面”、“一袋米”、“亿只鸡”工程，组建农产品质量安全监测中心，不断提高农产品优质率、转化增值率和市场占有率。扶持壮大优势产业和特色农业，巩固随州作为全省重要粮仓、优质畜禽产品生产基地的地位，加快建设全国最大的香菇标准化生产和加工出口基地。引导发展现代休闲观光农业，推进现代农业建设。探索建立农业生产经营服务新机制、新模式，不断提高农业组织化程度。

加快提升现代服务业。突破性发展文化旅游业，做精做美炎帝故里景区、擂鼓墩编钟景区、大洪山宝珠峰核心景区和徐家河休闲度假旅游区，力争“十二五”末年接待游客1500万人次，旅游综合收入120亿元。抢抓国家扩大内需政策机遇，加快发展生活性、生产性服务业，重点建设会展、研发、物流、职教四大区域服务中心，引导资源要素集聚，打造区域性物流节点城市。推进城市商贸业发展，加快向农村延伸步伐。建立健全现代金融体系，积极引进各类商业银行、创投、信托、证券、保险等金融机构，扩大村镇银行布点，实现最佳金融信用市和县市区全覆盖。

（二）全力推进项目建设，在增添发展后劲上实现新跨越。坚定不移地抓投资、促增长，统筹策划、实施一批事关发展大局的基础性、战略性、支撑性重大项目，力争固定资产投资年均增长25%以上，五年累计完成3000亿元。紧扣国家产业政策，结合随州实际，重点储备、争取和实施三类项目。一是谋划一批有利于调结构、促转型的新型工业化、城镇化项目。重点谋划50个投资过10亿元的工业项目，抓好食用菌精深加工，抓紧实施一批现代物流项目，力促中华农耕文明园、擂鼓墩大遗址保护等项目挤进国家和省里的笼子。二是谋划一批打基础、管长远的基础设施项目。重点实施麻竹高速随州西段、随信铁路、麻随铁路、月益铁路随州段和境内各干线公路等项目，将随州建成区域性交通枢纽城市。每年投资不低于10亿元，用于完善城市功能。加快实施水库除险加固、灌区续建配套与节水改造、中小河流治理工程。三是谋划一批惠民生、促和谐的生态环境、社会事业项目。重点实施府河、城市内河治理和污水处理等项目，加快推进大风口风电开发、广水核电和生物质能源等项目的前期工作，启动500千伏变电站建设。

（三）全力深化改革开放，在增强发展活力上实现新跨越。坚持把改革开放作为促进科学发展、跨越发展的强劲动力，着力破解发展难题，拓宽发展空间，赢得发展先机。

创新机制抓改革。围绕把随州打造成全省发展成本最低、审批事项最少、服务效率最高的城市，着力巩固和完善大部制成果。深化行政管理体制改革，进一步完善乡镇机构综合配套改革，建立农村公益性服务新机制。继续深化国有企业和公用事业单位改革。全面推进医药卫生体制改革。逐步扩大农村养老保险的覆盖面。

借力发展抓招商。坚持把招商引资作为经济工作的重中之重，以提升产业竞争力为目标，以推动企业战略重组为重点，承接沿海、对接武汉，加快建设产业转移工业园，优化政策措施，创新招商方式，加强协调服务，力争引进一批战略投资者和10个中国500强企业。

扶持龙头抓外贸。大力扶持壮大外贸出口主体，拓展出口市场，打造出口产业集群，力争到2015年，全市出口企业发展到400家。加快推进出口多元化战略，创建省级农副产品、汽车及零部件、电子信息产品出口基地。

（四）全力统筹城乡发展，在加快新型城镇化进程上实现新跨越。坚持走随州特色的新型城镇化道路，着力构建一主两翼、三足鼎立、多点布局的城镇化发展格局，确保“十二五”期末城镇化率达到50%以上，力争赶上全省平均水平。加快主城区向大城市迈进步伐。以提高宜居质量为根本，以完善城市公共设施为基础，以向东和向东南发展为主，继续拉开城市骨架，将城区面积拓展到80平方公里，使随州城逐步向功能齐全、宜居宜业的大城市迈进。打造各具特色的中等规模城市。加快推进随县新县城建设，努力建成在全省县域中具有鲜明特色和充满活力的新县城；支持广水城区向新区拓展，提高容纳空间和发展水平，朝着中等规模城市目标迈进。以更大的力度推进新型城镇化。加快城乡一体化建设步伐，力争曾都区成为全省示范区。按照人口、项目、产业逐步向城镇集中的要求，加快建设一批具有较强辐射带动力的重点镇、特色镇；坚持规划引领、产业带动、示范引导、一体化推进，提高新农村建设水平。

（五）全力保障和改善民生，在和谐随州建设上实现新跨越。以人民群众共享发展成果为取向，完善政策措施，加大投入力度，促进社会和谐。

扶持创业带动就业。继续实施积极的就业政策，坚持一手抓扩大就业，一手抓回归创业，保持全市就业形势基本稳定，力争“十二五”期间城镇新增就业10万人以上。

完善社会保障体系。在扩大覆盖范围、提高保障水平、提升统筹层次和加强制度衔接上下功夫，确保社保覆盖面达到95%以上。加大保障性住房和农村安居工程建设力度，解决低收入群体的住房难问题。提高贫困认定标准，增加扶贫投入，每年解决1万农村人口的脱贫问题。

全面发展社会事业。实施科教兴市和人才强市战略，大力培育高新技术企业和产业，推进科技创新和科技进步。进一步优化教育资源配置，调整义务教育学校布局，有序启动中小学标准化建设；普及发展学前教育，重视发展高中阶段教育，扶持发展职业教育，鼓励和规范发展民办教育，初步实现教育均衡发展。不断提高新农合保障水平，完善基层医疗设施建设。继续稳定低生育水平，全面提升人口素质。高度重视发展文化体育事业，加强文化遗产保护，合理利用文物资源，大力推进文化、旅游与经济的深度融合。

切实加强社会管理。推进依法治市进程，启动“六五”普法工作，进一步创新社会管理机制；完善社会治安防控体系，始终保持对各类刑事犯罪行为的严打高压态势。改进和加强信访工作，健全群众利益维护机制。不断完善各种应急预案，有效应对自然灾害、事故灾害和公共卫生、社会安全等突发事件，努力维护社会稳定。进一步提高综合减灾救灾能力，切实加强安全生产，有效防范和坚决遏制重特大事故的发生，加快和谐随州建设。

三、2011年的重点工作

2011年是实施“十二五”规划的基础之年、开局之年，做好今年的各项工作，意义深远，责任重大。必须坚持应急与谋远相结合，正确把握当前的经济形势、增强跨越式发展的信心和决心，正确把握今年经济工作的总体要求、突出工业兴市主战略，正确把握今年各项工作的奋斗目标，努力实现“十二五”开门红。

基于对发展形势的分析和判断，今年经济社会发展的主要预期目标是：地区生产总值增长12%以上，规模工业增加值增长18%以上，全社会固定资产投资增长30%以上，地方一般预算收入增长13%以上，外贸出口增长13%，城镇居民人均可支配收入增长11%，农民人均纯收入增长9%，社会消费品零售总额增长17%，居民消费价格涨幅控制在4%以内，单位生产总值能耗下降2%以上，化学需氧量等“四项约束性指标”全面完成省定目标。各项社会事业协调推进、加快发展。

上述目标是一个预期目标，是综合考虑宏观环境和我市有利条件及制约因素，兼顾当前和长远、需要和可能而提出来的。保持这样一个相当的增长速度，有利于释放现有的产能，有利于调整优化经济结构，有利于改善和保障民生。在实际工作中，我们将自加压力，自压担子，按照跨越式发展的要求，确定更高的工作目标来保证预期目标的全面完成，争取实现更好更快的发展。重点抓好八个方面的工作：

（一）推进工业兴市主战略，加速提升竞争实力。围绕建设“百亿企业、百亿产业和百亿园区”的目标，推进工业结构调整，加速扩张工业总量。

一是扶持壮大支柱产业。立足于构建总量规模大、集中度高、上下游产品配套、市场竞争力强的工业体系，充分发挥省部共建中国专用汽车之都的机遇，着力打造汽车产业服务平台，加快完善湖北省专用汽车质量检测中心建设，力争将其打造成国家级检测分中心。以恒天汽车、厦门重工、齐星车身、东风专汽、中国重汽等企业为龙头，不断提高技术装备水平和产品档次，扩大整车生产规模，加快开发关键零部件，打造汽车机械产业品牌。支持三友食品、神农生态、银丰面粉等企业产品精深开发、抢占市场份额，进一步提升支柱地位。鼓励引导纺织服装加工企业做大做强，重点支持钰马纺织、兴发纺织、澳门纺织工业园建设。大力发展生物医药、中成药、中药材和化工产业，提高产品质量，扩大生产规模。积极培育新兴产业，倾力支持多晶硅上下游项目及关联企业的引进和发展，加快有机硅新材料、液晶显示器、动力电池等产品的研发和产业化步伐，引导通讯电子、消费电子及半导体照明产业发展。支持广水建设中国风机名城。加大企业上市推进力度，滚动培育上市后备企业，重点支持 犇星化工、同星农业、泰晶科技、双剑公司等企业上市。

二是推进重点项目建设。加快在建和新上项目推进力度，突出抓好50个投资5000万元以上的工业项目，加快化工工业园、恒天汽车工业园、奥龙专用车工业园等重点项目建设进度。积极对上争取支持，大力实施重大技改工程，重点抓好齐星车身生产线扩能改造、全力公司制动器等50个过千万元工业技改项目，增强工业发展后劲。

三是加快园区平台建设。坚持规划引领，在开发上实行“整合、优化、扩容”三同步，在布局上实现“集聚、集群、集约”三提高，在功能上实现“政务、商务、服务”三配套，推进园区产业集群发展。支持市开发区拉开框架，优化软硬件环境，加快华裕工业园建设，尽快形成 “一区多园”的发展格局，打造全市工业核心增长极。支持各县市区开发区配套完善、提档升级。因地制宜推进各类特色工业园区建设，打造一批工业强镇。

四是大力培育市场主体。配套完善推进工业兴市相关措施，深化一企一策、对口帮扶机制，选择一批具有成长潜力的中小企业，优化配置各类生产要素，加速推进 “小进规”步伐，确保净增销售收入2000万元以上的规模企业30家。积极对上争取科技项目，加快科技型企业孵化器建设，新增高新技术企业5家以上。加强创业园建设，深入开展市场主体增量行动，鼓励引导全民创业，力争新增各类市场主体15000家。

五是加强企业家队伍建设。工业兴市，企业是主战场，企业家是主力军。政府将以更加开明的姿态，大力营造尊重企业家、尊重创业者的良好氛围。进一步加强与企业的互动，充分发挥各类商会、协会的桥梁纽带作用，积极解决人才引进中的个性化问题，着力培育企业领军人物。同时，也希望企业以更加开放的胸怀，加快引进战略合作者和高端人才，消化吸收先进成果，力争实现外延扩大再生产和内涵技术改造的双突破，创建一批名牌产品和驰名、著名商标。

（二）夯实“三农”发展基础，加快新农村建设步伐。始终把“三农”工作作为各项工作的重中之重，坚持以工业化的理念谋划农业，按照“三化同步”和“两增同步”的要求，加速推动传统农业向现代农业转型。

一是扶持壮大特色农业。按照优质、高产、安全和高效的要求，突出特色、规模、品牌和效益，引领特色农业发展。进一步调整优化农业结构，在稳定发展粮棉油的同时，突破性发展特色产业，确保食用菌增产10%以上；大力实施新一轮“菜篮子”工程，高标准落实菜地最低保有量，进一步扩大马铃薯、菜豌豆、大蒜、优质桃、油茶、中草药板块面积和优质肉牛、三元猪、山羊、三黄鸡养殖规模。积极推行标准化生产，加强“三品一标”认证和监管。大力发展农产品加工业，推进农业产业化经营，鼓励引导龙头企业加快对外重组和转型升级步伐，构建完整产业链条，重点培育一批“农字号”大型龙头企业和企业集团。

二是多途径增加农民收入。深入开展科技下乡、技能培训等活动，大力发展农民专业合作组织，带动农民科学致富、创业致富。加快完善机耕道、维修站等配套设施，提高农业生产机械化程度。建立健全农业服务体系，总结推广三里岗“农户联保”模式。深入开展“创绿色家园，建富裕新村”活动，组织实施退耕还林、低产林改造、生态林等工程，让农民得到实惠。大力转移农村劳动力，不断提高工资性收入占农民收入的比重。突出抓好整村推进扶贫和老区片区开发。

三是下大力夯实农业基础。抢抓国家农业基础建设重点向小农水、小水库等方面倾斜的政策机遇，积极向上争取项目，掀起新一轮农田水利建设高潮。完成氵厥水河厉山段、漂水河殷店段治理任务，推进随中和广水黑花飞灌区建设，实施50座重点小型水库除险加固，大力开展国家小型水利重点县和省现代农业重点县建设，完成广水市国家安全饮水工程示范市建设任务。巩固新农村建设成果，加快推广实验区的成功经验，尽快形成辐射效应。

四是突破性发展县域经济。坚持宜工则工、宜农则农、宜游则游，积极推进县域经济发展，鼓励产业、企业向园区聚集，摒弃“村村点火，户户冒烟”的零散式、资源浪费式的乡镇工业发展模式。不断创新工作机制，把调动乡镇发展和县域发展的积极性有机结合起来，凡是乡镇引进项目进入市、县园区的，其经济收益、统计指标和政绩考核计入乡镇，进一步调动各地的积极性和创造性。通过不懈努力，力争曾都区尽快重返全省县域经济10强、广水市进入第一方阵、随县获得争先进位奖。

（三）继续深化改革开放，拓展外贸增长空间。进一步加大招商引资力度，积极筹备参加第六届中博会、鄂港粤经贸洽谈会等招商活动，突出产业招商，注重以商引商，激励部门招商，鼓励全民招商，进一步提高招商工作的科学化、专业化水平，着力引进一批中国500强和“中”字号大型企业，确保引进内联资金65亿元，实际利用外资5000万美元以上。在抓好招商项目的同时，继续加大对上争取力度，确保争取无偿资金4亿元以上。落实项目建设责任制和“一条龙”、“直通车”制度，为项目落户提供保姆式服务。加快实施“走出去”战略，巩固亚洲传统市场，大力开发美国、欧盟、澳洲等发达国家市场，加快拓展东欧、非洲及南美洲等新兴市场，培育壮大出口骨干企业，尽快将多晶硅等高新产品推向国际市场，着力构筑农副产品、机械产品、电子纺织产品三足鼎立的出口格局，努力实现出口8亿美元以上，进一步巩固随州外贸大市的地位。不断深化各项改革，重点抓好国有企业改革收尾工作，进一步深化财税体制改革，继续推进事业单位改革，加强国有资产监管，推进集体林权制度配套改革，进一步增强经济社会发展的体制机制活力。

（四）完善城市功能布局，统筹城乡一体发展。紧盯向大城市迈进的目标，拉框架、扩规模、提档次、升品位，实现“组团、互通、成片”发展。在城市建设上，主城区重点实施“十大工程”：一是道路完善配套工程。加快老城区道路改扩建，延伸汉东路，改造青年路东段；加快新城区道路完善配套，新建编钟大道北端、清河东路；启动青年路大桥和程力大桥建设。二是“一河两岸”景观工程。延伸白云大道风光带，形成完整的滨湖循环走廊，进一步提高绿化、美化、亮化的档次，提升城市品位，为市民提供良好的休闲场所。三是城市内河整治工程，按照“防洪、雨污分流、生态景观、休闲”的原则，力争用三至五年时间完成。今年重点实施花溪河治理工程。四是进一步加快污水管网建设，完善城市污水收集系统；启动望城岗污水处理厂二期工程；加快实施垃圾清洁填埋工程，建设城南垃圾处理场。五是启动城东新区季梁公园建设，完成人工湖和文化展示园工程，逐步完善建筑、道路和绿化景观配套建设。六是结合棚户区改造，启动历史文化街改造工程，着力打造彰显随州特色的历史文化街区。七是规划建设传媒中心。八是完成何店220千伏变电站主体工程。九是新建滨湖体育场，建成城区市民体育健身工程10处以上。十是建设清洁能源工程，完善城区天然气管网，新建2座加气站，基本实现城区市民和企业用上中亚优质天然气。在城市管理上，推进新一轮“三城同创”，启动并加快国家级园林城市、森林城市、省级环保模范城创建工作。进一步完善城管制度，实行网格式、精细化管理，建立街道、社区、单位和市民共同参与的长效机制。严格规划审批程序，确保规划的严肃性，始终坚持逢违必拆的高压态势，严厉打击违法建设。实施疏堵保畅工程，突出抓好城区交通秩序管理，综合整治违章占道、违禁行驶、乱停乱靠、随意横穿马路等顽症；大力发展城市交通，优化公交线路，完善智能交通系统。在村镇建设上，继续推进“楚天杯”创建和“百镇千村”工程，打造集镇建设新亮点；加强村庄环境整治，建成一批 “宜居村庄”；加快交通设施建设，确保麻竹高速大随段年底竣工通车，麻竹西段建设、广水市中心客运站和物流中心、随县中心客运站和农副产品冷链物流中心等项目如期启动。

（五）加快发展文化旅游，扩大城乡消费需求。围绕建设全国知名的旅游目的地，建立健全旅游强市建设推进机制，坚持政府主导、企业主体、部门配合、社会参与，启动一批旅游项目建设，建好重点景区景点，打造“拜炎帝始祖、听编钟神曲、看曾侯古墓、泡玉龙温泉、游灵山秀水”精品旅游线路。整合博物馆、曾侯乙墓遗址资源，加快擂鼓墩景区建设，进一步擦亮“中国编钟之乡”文化品牌；持续推动大洪山整体开发，开工建设门景区项目和景区内循环路改造，推动桐柏山综合开发取得实质性进展；加快三潭景区、徐家河休闲度假区和尚市桃花园景区建设，完成洛阳千年银杏谷景区配套工程。继续高规格、高标准办好炎帝故里寻根节，将其打造成全国重要的节会品牌。广泛开展宣传促销，创作一批以炎帝、编钟为主题的文艺作品。积极拓展旅游客源市场，力争接待国内外游客650万人次，实现旅游综合收入47亿元。加大对旅游企业和旅游人才的培育扶持力度，抓好旅游名镇名村创建活动。加快发展商贸服务业，鼓励商贸龙头企业扩张规模，支持建设物流配送中心。大力发展连锁经营、电子商务、网络购物、专业配送等新型业态。继续实施“万村千乡市场工程”和“新网工程”，建设一批规范的超市和农家店。大力促进农村消费，简化汽车摩托车、家电下乡和农机购置补贴程序。积极培植城市消费热点，引导发展服装、家具、建材等专业市场，加强餐饮酒店、教育培训、医疗保健等服务场所规范化建设和管理。加大协调力度，执行好差别化住房信贷政策，大力遏制投机性购房；调整优化商品房供应结构，健康稳定发展房地产市场。强化价格监管职能，审慎出台政府定调价项目，努力保持市场价格总体水平基本稳定。切实加强各类专业市场监管，保障市民消费安全。

（六）做好财税金融工作，增强支撑保障能力。认真落实积极的财政政策，充分调动各地培植财源、组织收入的积极性，努力保持财税收入稳定增长。进一步优化支出结构，把更多的财力用于支持工业项目、社会建设和民生改善。推进财政精细化管理，不断提高资金使用效益。加强政府性债务管理，有效防范和化解财政风险。进一步优化金融生态环境，巩固政银企战略合作成果，促进银企项目对接。加快社会信用体系建设，组建主体多元化、运作市场化的担保机构，扶持发展小额贷款公司，有效缓解企业特别是中小企业资金瓶颈制约。

（七）大力发展各项事业，促进和谐社会建设。牢固树立“改善民生就是发展、就是政绩”的理念，统筹经济社会协调发展。

抓好就业和社会保障工作。开展教育扶贫、技术济困活动，做好政策服务、创业培训工作，扩大小额担保贷款资金使用范围，切实抓好高校毕业生、农民工、复退军人和下岗失业人员的就业再就业。加大社会保险扩面征缴力度，确保五项保险参保总数突破100万人次，稳步提高社会保险待遇标准；巩固新农保试点成果，扩大被征地农民养老保险参保范围。不断拓宽住房公积金制度覆盖面，强化资金监管，进一步扩大贷款规模，努力实现应贷尽贷。

协调发展社会事业。加大教育投入，优化整合教育资源，继续实施 “校安工程”，新建扩建6所城区初中、小学和幼儿园，进一步改善办学条件；定向培养技能人才，提升随州职教品牌。不断提高新农合保障能力，积极推进基本药物制度，加快县乡村三级基层医疗服务能力建设；进一步完善城乡贫困群众大病医疗救助制度，促进公共卫生服务均等化；切实增强应对突发公共卫生事件的能力。建立健全社会救助制度，继续实施乡镇福利院改扩建工程。进一步完善计生服务体系，坚决遏制违法生育，严厉打击“两非”行为，严格控制出生人口性别比。加强国土资源管理，依法依规利用土地，努力保持耕地占补平衡。强化招投标综合监管，启动建设公共资源交易市场。全面加强污染防治和环境管理，确保环保模范城创建工作取得实质性进展。重视发展老龄、残疾人事业，切实保障妇女和未成年人合法权益。依法管理宗教事务，进一步做好统计、国调、审计、司法、档案、保密、对台、史志、气象、水文、外事侨务、慈善、红十字会和无偿献血等工作。

深入推进社会主义精神文明建设。突出社会主义核心价值体系建设，以“文明让生活更美好”为主题，大力开展新一轮文明城市创建活动。加强文化阵地建设，完成农民体育健身工程50处以上，扩大广播电视覆盖面，深入开展打黄扫非和新闻出版市场、文化娱乐场所、网吧、电子游戏室专项整治，大力保护文物和非物质文化遗产，筹备好市第二届体育运动会。做好人民防空和防震、减灾、救灾工作，以新一轮“双拥模范城”验收命名为契机，加强国防动员、民兵预备役应急能力建设，做好军队转业干部、复退军人的安置和优抚工作，增进军政军民团结。

全力维护社会稳定和谐。全面推行社会稳定风险评估机制，不断提高危机管理、应急处置和抗风险能力。加强社会治安综合治理，重拳打击违法犯罪，重点打击“两抢一盗”等犯罪行为，坚决取缔各种传销活动。坚持严格执法、调解优先，以信访涉法涉诉为重点，进一步加强矛盾纠纷的排查和调处，深入开展领导干部“四访”活动，着力化解信访积案。巩固完善安全生产保障制度，切实落实企业主体责任，深入开展重点行业、重点领域的安全监管和专项整治，有效防止重特大事故的发生。

（八）加强执行力建设，树立政府新形象。新的形势和任务对政府工作提出了新的更高的要求，我们将以执行力建设为重点，切实做到依法行政、科学理政、高效施政、廉洁从政。

严格依法办事，规范决策程序。认真执行人大决议，自觉接受人大监督，加强同人民政协、民主党派、工商联和无党派人士的民主协商，认真办好议案、建议和提案。坚持科学决策、民主决策，完善听证、论证和专家咨询制度，建立健全公众有序参与的体制机制，努力用优良的决策奠定可靠的执行基础。办好政府门户网站，加大政务公开、办事公开、决策公开力度，积极接受新闻舆论和社会公众的监督。

推进管理创新，提高行政效能。加强公务员队伍建设，不断提高工作能力和水平，细化分解目标任务，建立完善工作推进、协调、落实、反馈和评估制度，全面推行目标管理和岗位绩效考评，做到程序服从效率、效率倒逼程序，加快行政提速。创新行政服务运行机制，全面落实“三集中三到位”；加快电子政务建设，扩大办公自动化使用范围，逐步普及网上申报、网上审批、网上缴费。

加强反腐倡廉，严肃政风政纪。认真贯彻执行党风廉政建设责任制，巩固“十个全覆盖”建设成果，把反腐倡廉落实到公共资源配置、公共资产交易、公共产品生产等权力运行的各个环节。加强领导干部经济责任审计，严格执行财经纪律，坚持厉行节约，勤俭办事，降低行政成本。进一步严肃工作纪律，转变工作作风，大力弘扬不事张扬、真抓实干、敢于争先的精神，深入开展行风评议，切实用硬措施优化软环境。

坚持为民执政，办好十件实事。为人民群众办好事、办实事，是各级政府应尽的职责。今年我们将集中财力，继续为市民办好十件实事：（1）鼓励创业带动就业，发放小额担保贷款3000万元以上，帮扶1000名有创业愿望和创业能力的劳动者成功创业；城镇新增就业1.58万人，开发公益性就业岗位1000个。（2）筹资1.8亿元，用于全市义务教育阶段学生“两免一补”和5.8万高中阶段贫困学生的资助。（3）加大政府投入力度，巩固完善曾都区基本药物制度改革成果，加快推进广水市实施国家基本药物制度、确保药品实现零差价销售，缓解群众看病贵的问题。（4）启用市儿童福利院，完善配套设施；启动建设市残疾人托养中心；改造完善3个乡镇福利院；实行惠民殡葬，全部免除城乡低保户、农村五保户、重点优抚对象等特困群体的基本殡葬费用。（5）新增投资2亿元，加快农村电网升级步伐，完成730个台区改造，惠及620个行政村、1.68万个家庭。（6）全面完善20户以上自然村广播电视村村通工程，高标准完成500个点的20户以下自然村村村通建设任务。（7）新增廉租住房680套，新增发放廉租住房货币化补贴1300户，新建公共租赁住房1000套。（8）继续采取以奖代补的方式，加快城区社区标准化建设；继续实施主城区背街小巷改造工程，确保今年基本完成。（9）投资5000万元，实施智能监控改造工程，完成指挥平台建设，实现主城区主要道路、社区、出城口视频监控全覆盖。（10）解决农村7万人口饮水安全问题；继续加大先觉庙水源地保护力度，启动先觉庙供水复线改造工程，确保市民喝上更加安全更加放心的饮用水。

各位代表，今天的随州，正处在充满活力、彰显魅力、积聚实力的重要阶段，宏伟的蓝图引领我们开拓进取，美好的未来激励我们奋力拼搏。让我们在中共随州市委的坚强领导下，在市人大、市政协的监督支持下，紧紧依靠全市人民，深入贯彻落实科学发展观，顺势而为，自我加压，开拓进取，扎实工作，奋力谱写随州科学发展、跨越发展、和谐发展的新篇章！
